# The lipid substrate preference of CETP controls the biochemical properties of HDL in fat/cholesterol-fed hamsters

**DOI:** 10.1016/j.jlr.2021.100027

**Published:** 2021-01-27

**Authors:** Richard E. Morton, Daniel Mihna, Yan Liu

**Affiliations:** Department of Cardiovascular and Metabolic Sciences, Lerner Research Institute, Cleveland Clinic Foundation, Cleveland, OH, USA

**Keywords:** cholesteryl ester transfer protein, substrate specificity, HDL metabolism, hepatic gene expression, hepatic cholesterol, reverse cholesterol transport, cholesterol efflux, LCAT, SRBI, triglyceride, ApoF, apolipoprotein F, CE, cholesteryl ester, CEth, cholesteryl ether, CETP, cholesteryl ester transfer protein, CHO, Chinese hamster ovary, DiI, 1,1′-dioctadecyl- 3,3,3′,3′-tetramethylindocarbocyanine perchlorate, FC, free cholesterol, FPLC, fast-protein liquid chromatography, PL, phospholipid, TC, total cholesterol, TG, triglyceride, RCT, reverse cholesterol transport

## Abstract

Cholesteryl ester transfer protein (CETP) modulates lipoprotein metabolism by transferring cholesteryl ester (CE) and triglyceride (TG) between lipoproteins. However, differences in the way CETP functions exist across species. Unlike human CETP, hamster CETP prefers TG over CE as a substrate, raising questions regarding how substrate preference may impact lipoprotein metabolism. To understand how altering the CE versus TG substrate specificity of CETP might impact lipoprotein metabolism in humans, we modified CETP expression in fat/cholesterol-fed hamsters, which have a human-like lipoprotein profile. Hamsters received adenoviruses expressing no CETP, hamster CETP, or human CETP. Total plasma CETP mass increased up to 70% in the hamster and human CETP groups. Hamsters expressing human CETP exhibited decreased endogenous hamster CETP, resulting in an overall CE:TG preference of plasma CETP that was similar to that in humans. Hamster CETP overexpression had little impact on lipoproteins, whereas human CETP expression reduced HDL by 60% without affecting LDL. HDLs were TG enriched and CE depleted and much smaller, causing the HDL3:HDL2 ratio to increase threefold. HDL from hamsters expressing human CETP supported higher LCAT activity and greater cholesterol efflux. The fecal excretion of HDL-associated CE in human CETP animals was unchanged. However, much of this cholesterol accumulated in the liver and was associated with a 1.8-fold increase in hepatic cholesterol mass. Overall, these data show in a human-like lipoprotein model that modification of CETP's lipid substrate preference selectively alters HDL concentration and function. This provides a powerful tool for modulating HDL metabolism and impacting sterol balance in vivo.

Cholesteryl ester transfer protein (CETP) impacts human lipoprotein metabolism by facilitating the transfer of cholesteryl ester (CE) and triglyceride (TG) between lipoproteins (1, 2, 3). Whether CETP transfers CE or TG is strongly influenced by the relative abundance of these lipids in the participating lipoproteins. When transferring lipids between VLDL (a TG-rich lipoprotein) and LDL or HDL (CE-rich lipoproteins), CETP promotes the net transfer of TG from VLDL into LDL and HDL and the net transfer of CE in the reverse direction. CETP activity, in effect, drives the ratio of CE and TG in plasma lipoproteins toward equilibrium.

Another factor influencing the extent to which CETP modifies lipoproteins is the relative preference of CETP itself for CE versus TG as a substrate. Mutations of single amino acid residues in CETP are sufficient to change its preference for CE versus TG as a substrate (4), which alters the redistribution of these lipids between TG- and CE-rich lipoproteins (5). This suggests that the equilibrium point for CETP-dependent redistribution of CE and TG among lipoproteins is, at least in part, controlled by the preference of CETP for its different lipid substrates. This substrate preference can also be altered by antibodies to CETP (5), showing that this property of CETP can be manipulated.

CETPs from different species have unique preferences for CE versus TG as substrate (6). To examine whether altering the substrate preference of plasma CETP impacts lipoprotein metabolism in vivo, we recently expressed human CETP in hamsters. Human and hamster CETPs have greatly different preferences for CE versus TG as a substrate. Those studies in chow-fed hamsters clearly demonstrated that modification of the substrate specificity of plasma CETP drives large changes in the levels and composition of lipoproteins, especially those of HDL. Whether this preferential impact on HDL simply reflects the fact that HDL is the major lipoprotein in these chow-fed animals is not known. To address this question, and to better understand how altered CETP substrate specificity might impact lipoprotein metabolism in humans, here we extend our previous proof-of-concept studies to a more human-like model. In this fat-fed model, hamsters have total plasma cholesterol levels similar to those of humans and LDL is the major cholesterol-carrying lipoprotein in plasma. Of importance, this diet also raises VLDL several-fold to levels commonly observed in humans. VLDL levels may be rate limiting for CETP activity in vivo (7, 8). In this model, HDL continued to be the focus of changes caused by modifying CETP's substrate specificity. However, novel alterations in the physical and functional properties of this lipoprotein and in hepatic cholesterol balance were induced.

## Methods

### CETP adenoviruses

A vector containing human CETP cDNA (M30185.1) was purchased from Open Biosystems (Pittsburgh, PA). The cDNA for golden Syrian hamster CETP (*Mesocricetus auratus*, XM_005079728.2) was synthesized by Gen-Script (Piscataway, NJ). In vivo quality, recombinant E1/E3-deleted adenovirus (serotype 5) constructs containing the CMV promoter alone (Ad-null), hamster CETP (Ad-haCETP), human CETP (Ad-huCETP) were custom synthesized by Vector Biolabs (Malvern, PA).

### Animals

Male golden Syrian hamsters (101–110 g, ∼7 weeks old) were purchased from Charles River Laboratories (Wilmington, MA). A jugular vein catheter was surgically placed and exteriorized dorsally. Hamsters were allowed to recover approximately 1 week. Studies were initiated by injecting adenovirus and switching animals to a high-fat/cholesterol diet (day 0). Jugular vein catheters were flushed with sterile saline followed by injection of adenovirus (2.5–5 × 10^9^ pfu), a second saline flush, and then heparin/glycerol catheter lock solution (Braintree Scientific, Inc., Braintree, MA). Animals received ad libitum a standard chow diet supplemented with 0.12% cholesterol and 20% hydrogenated coconut oil (Envigo, Madison, WI) for the duration of the study. On day 4, the reverse cholesterol transport assay was initiated (described below), and the experiment was terminated on day 6. In separate studies, it was determined that plasma levels of total cholesterol and of individual lipoproteins, and the adenovirus-mediated increase in plasma human or hamster CETP levels, were essentially the same on day 4 and day 6. The Cleveland Clinic's Institutional Animal Care and Use Committee approved all animal studies.

Some animals were excluded from the final data set. To minimize the effects of hyper-responsiveness of individual animals to the high-fat/cholesterol diet, four animals (two Ad-null, one Ad-haCETP, and one Ad-huCETP) with plasma cholesterol > 250 mg/dl, which is more than two standard deviations above the mean for each group, were excluded. Other animals were excluded because the adenovirus injection failed to significantly alter plasma CETP levels. These included five Ad-haCETP animals, in which plasma CETP levels were not different from control animals. Since the plasma concentration of lipoproteins and physicochemical properties of LDL and HDL isolated from these animals were indistinguishable from those of Ad-null control animals, this group was not studied further. Two Ad-huCETP animals were excluded owing to very low huCETP expression (≤10% of the Ad-huCETP mean). A subset of Ad-huCETP animals with very high human CETP levels is reported separately.

### Reverse cholesterol transport

To measure reverse cholesterol transport (RCT) from HDL, on day 4 after adenovirus injection the jugular vein catheters were flushed with saline, injected with ^3^H-CE HDL (∼125 μg protein, 2 μCi ^3^H), followed by a second saline flush and catheter lock solution. Animals were transferred to cages with wire-bottom platforms to facilitate feces collection. After 48 h (day 6), animals were euthanized. Blood, liver, and feces were collected and processed, and ^3^H was quantified as previously described (9). HDL for RCT assays was labeled with [1,2-^3^H(N)]cholesteryl oleate (PerkinElmer, Inc., Waltham, MA) as previously described (10).

### Quantification of CETP mass and activity in hamster plasma

Hamster and human CETPs in plasma were quantified as previously described (9). Briefly, hamster plasma was reacted with TP2 anti-CETP antibody (Ottawa Heart Institute, Ottawa, ON, Canada) and immune complexes were captured on M-280 sheep anti-mouse IgG magnetic Dynabeads (Invitrogen, Carlsbad, CA). Human and hamster CETPs were separated on 8% SDS-PAGE gels (Invitrogen) and transferred to PVDF. CETPs in samples and standards were detected with TP2 antibody followed by a goat anti-mouse IgG HRP secondary antibody (11). Bands were visualized by Western Lightning Plus ECL reagent (Perkin-Elmer Life Sciences). Chemiluminescence was captured on a digital imager (GE Healthcare, Marlborough, MA) and quantified by ImageJ (https://imagej.nih.gov/ij/). CETP levels are expressed relative to that contained in Ad-null hamsters.

The CETP activity in hamster plasma was quantified as previously described (10). This assay measured the capacity of plasma samples to promote the transfer of radiolabeled TG and CE from LDL to HDL under conditions where the effect of endogenous lipoproteins on CETP activity is minimized. For these assays, LDL was doubly labeled with [9,10-^3^H(N)]-triolein and cholesteryl-[1-^14^C] oleate (PerkinElmer, Inc.) by a dispersion method (12).

### Lipoprotein analysis

All physicochemical analysis and functional assays of hamster lipoproteins were performed on lipoproteins isolated from plasma by sequential ultracentrifugation (13). For some functional assays, isolated LDL and HDL were adjusted to 10% sucrose (14, 15) and stored at −20°C for future analysis. The protein concentration of isolated lipoproteins was measured by a modification of the Lowry *et al.* method (16) with BSA as the standard. Total cholesterol (TC), free cholesterol (FC), and TG were quantified by enzyme-based kits from Thermo Fisher Scientific (Waltham, MA) (TC, TG) and Wako Diagnostics Inc. (Mountain View, CA) (FC). CE was calculated as TC minus FC times 1.69 to adjust for the fatty acid contained in this molecule. Phospholipid (PL) phosphorus was determined chemically (17). HDL particle size distribution was determined by native gradient gel electrophoresis (18, 19).

To quantify the distribution of cholesterol among plasma lipoproteins, fresh plasma was fractionated by fast-protein liquid chromatography (FPLC) using tandem Superose 6 HR columns (GE Healthcare, Piscataway, NJ) (20, 21). Cholesterol in the eluant was continuously measured on-line. VLDL, LDL, and HDL peaks were identified based on the elution profile of hamster lipoproteins isolated by ultracentrifugation. Relative to Ad-null plasma, the cholesterol recovery from FPLC was 106 ± 6% and 107 ± 13% for Ad-haCETP and Ad-huCETP groups, respectively. For all plasma samples, the sum of cholesterol contained in VLDL, LDL, plus HDL peaks was highly correlated with the amount of plasma cholesterol applied (r = 0.94).

### Receptor-mediated LDL uptake

For each adenovirus group, LDL isolated from four to six animals were pooled and labeled with 1,1′-dioctadecyl-3,3,3′,3′-tetramethylindocarbocyanine perchlorate (DiI) (MilliporeSigma, St. Louis, MO) (22, 23) and reisolated by ultracentrifugation within their original density limits. HepG2/C3A cells [ATCC CRL10741, a derivative of HepG2 (ATCC HB8065)] were grown to 80% confluency in DMEM containing 10% FBS, then incubated overnight in DMEM containing 10% lipoprotein-deficient human plasma to upregulate LDL receptor expression. Subsequently, triplicate wells were incubated with the indicated amount of DiI-LDL in the same media. After 5 h, cells were washed twice with 0.4% BSA in PBS and three times with PBS. Cells were lysed with RIPA buffer and lysate DiI was quantified by fluorometry (520 nm ex, 580 nm em). Lysate protein was determined by BCA assay (Thermo Fisher Scientific). Receptor-independent uptake of DiI LDL, determined in the presence of 40-fold excess unlabeled LDL, was subtracted. *Km* and *V*_*max*_ values were determined from Michaelis-Menton plots.

### SRBI-mediated HDL CE uptake

The pooled HDL was labeled with the nondegradable CE analog, ^3^H-cholesteryl hexadecyl ether (^3^H-CEth) (Perkin-Elmer, Inc.), by the transfer of ^3^H-CEth from phosphatidylcholine/cholesterol liposomes (24). LDL-receptor-deficient Chinese hamster ovary (CHO) ldlA7 cells expressing mouse SRBI were a gift from Dr Monty Krieger (MIT) (25). Cells were maintained in Ham's F-12 media supplemented with 2 mM glutamine, 5% FBS, and 0.25 mg/ml G418 (Thermo Fisher Scientific). Cells at 80% confluence were incubated with ^3^H-CEth-labeled HDL in Ham's F-12 media containing 0.5% BSA. After 5 h, cells were washed twice with PBS and then chased for 30 min at 37°C in media containing 0.5% BSA and 100 μg/ml unlabeled HDL. Subsequently, cells were washed three times with PBS and lysed with RIPA buffer. The ^3^H-CEth content of the lysate was determined by liquid scintillation counting. The lysate protein was determined by the BCA method (Thermo Fisher Scientific). Receptor-independent uptake of HDL ^3^H-CEth, determined in the presence of 40-fold excess unlabeled HDL, was subtracted from reported values. ^3^H-CEth uptake by control ldlA7 cells not expressing SRBI was ∼10% of that in cells expressing this receptor.

### HDL support of LCAT activity

For each adenovirus group, HDLs from three animals were pooled. Pools were combined with ^3^H-FC ([1,2-^3^H(N)]-cholesterol, PerkinElmer, Inc.) and incubated overnight at 4°C. For the LCAT assay, ^3^H-FC HDL was incubated ± 50 μl lipoprotein-deficient human plasma as an LCAT source, 60 μl of 2.5% FFA-free BSA, and 0.9% NaCl, 0.02% NaN_3_, and 0.7 mM EDTA, pH 7.4 buffer to a final volume of 600 μl. Triplicates were incubated at 37°C for 0 h (blank) or 1 h. CE synthesis was stopped by the addition of 2 ml ethanol. Lipids were extracted (26) and fractionated by TLC (hexanes:diethyl ether:acetic acid, 70:30:1). The ^3^H content of FC and CE bands was determined by liquid scintillation counting, and the fraction of total ^3^H recovered in the CE band was calculated. LCAT activity contained in the HDL samples, determined from samples incubated without exogenous LCAT, was subtracted from these values. This corrected fraction, times the HDL FC (μg) contained in the assay, gave the amount of esterified cholesterol formed.

### HDL-facilitated cholesterol efflux

Cholesterol efflux was measured as previously described (9). Briefly, RAW 264.7 mouse macrophages (TIB-71, American Type Culture Collection, Manassas, VA) were labeled with ^3^H-cholesterol and then incubated overnight ± 0.3 mM 8-bromo cAMP (MilliporeSigma) to upregulate *ABCA1* expression. Subsequently, washed cells were incubated in the same medium containing either ApoB-depleted serum (27) or isolated HDL as a cholesterol acceptor. After 4 h, the medium was removed and centrifuged to remove cell debris. Cells were solubilized with RIPA buffer. ^3^H in both fractions was quantified by scintillation counting. ABCA1-dependent efflux was determined from the difference between the total efflux (plus 8-bromo cAMP) and ABCA1-independent efflux (minus 8-bromo cAMP) cells.

### mRNA qPCR

Liver tissues were homogenized by a Tissuelyser II (Qiagen, Germantown, MD). Total RNA was extracted using TRIzol reagent (Invitrogen, Carlsbad, CA). First-strand cDNAs were synthesized using random primers and reverse transcriptase (Promega, Madison, WI). qPCR was performed using Power SYBR™ Green PCR Master Mix (Thermo Fisher Scientific) and a StepOnePlus RT PCR System (Life Technologies Corp.). qPCR primers are shown in supplemental Table S1. mRNA values were normalized to *ACTB*. Gene expression was calculated using the 2^-ΔΔCT^ method (28) and reported relative to Ad-null cells.

### Other analytical methods

To quantify hepatic cholesterol, liver homogenates were saponified with 2% ethanolic KOH and lipids extracted (26). The extract cholesterol was measured by a ferric chloride method (29). Bile acids in liver homogenates were measured by an enzymatic assay (Cell Biolabs, Inc., San Diego, CA). Western blots on liver homogenates were performed as generally described above for CETP to measure LDLr (3839-100, Biovision Inc., Milpitas, CA) and SRBI (NB400-104, Novus Biologicals, Centennial, CO). Beta-actin was used as the loading control (Novus Biologicals).

For statistical analysis between two groups, an unpaired *t*-test was used (Instat 3, GraphPad Software, San Diego, CA). *P* values < 0.05 were considered statistically significant. Statistical analysis involving comparisons between multiple groups was performed by one-way ANOVA with Tukey post test. When group variances were not equal according to Bartlett's test, multiple comparison tests were performed by Welch and Brown-Forsythe ANOVA with Dunnett's T3 post test (Prism 8, GraphPad Software, San Diego, CA). Adjusted *P* values < 0.05 were considered significant.

## Results

### CETP expression

Hamsters received intravenous control adenovirus (Ad-null) or an adenovirus expressing hamster (Ad-haCETP) or human (Ad-huCETP) CETP at the start of the experiment. After 6 days on a high-fat/cholesterol diet, plasma CETP mass was determined. Ad-haCETP increased plasma CETP by 70% above null controls (Table 1). In animals receiving Ad-huCETP, two different levels of human CETP expression were observed. In the first group, designated huCETP, the sum of hamster CETP + human CETP in the plasma of these animals was similar to the elevated CETP levels present in haCETP animals. The expression of human CETP in these animals also reduced endogenous hamster CETP levels, resulting in a plasma ratio of human CETP to hamster CETP of 3.6:1. In the second Ad-huCETP group, designated Hi huCETP, the expression of human CETP was threefold higher than in huCETP animals (Table 1).

**Table 1:** Plasma CETP mass and activity

Ad Group	Relative CETP Mass			Plasma Lipid Transfer Activities
	Hamster	Human	Total	CE/TG
Null (11)	1.00 ± 0.08	-	1.00 ± 0.08	0.10 ± 0.01
haCETP (10)	1.69 ± 0.17^b^	-	1.69 ± 0.17^b^	0.10 ± 0.01
huCETP (8)	0.29 ± 0.03^b^^,^^c^	1.05 ± 0.08	1.34 ± 0.10	1.34 ± 0.03^b^^,^^c^
Hi huCETP (5)	0.51 ± 0.06^a^^,^^c^	3.01 ± 0.22^d^	3.52 ± 0.25^b^^,^^c^^,^^d^	1.46 ± 0.06^b^^,^^c^

Ad, adenovirus.

Plasma CETP mass, relative to that present in Ad-Null animals, and TG and CE transfer activities in plasma were measured as described in the Methods. Mean ± SEM of indicated group sizes

a *P* < 0.05 versus null.

b *P* < 0.01 versus null.

c *P* < 0.01 versus haCETP.

d *P* < 0.01 versus huCETP.

Hamster CETP prefers TG over CE as a substrate, whereas human CETP prefers CE over TG. CE and TG transfer activities were measured to assess the impact of human CETP expression on the ratio of these two transfer activities in plasma. This ratio increased from ∼0.1 in animals expressing only hamster CETP to a value of 1.34 in huCETP animals (Table 1). This CE/TG preference is similar to the 1.5 ratio determined for purified human CETP in the same assay. Thus, although haCETP and huCETP animals have similar total plasma CETP levels, they are functionally distinctive. Although Hi huCETP animals have much higher human CETP levels than huCETP animals, this had no significant effect on the ratio of CE to TG transfer activities in plasma. Therefore, compared with huCETP animals, Hi huCETP animals report the effects of excess CETP expression without a measurable change in circulating CETP's substrate preference.

### Effect of CETP on plasma total and lipoprotein cholesterol

**Fig. 1:**
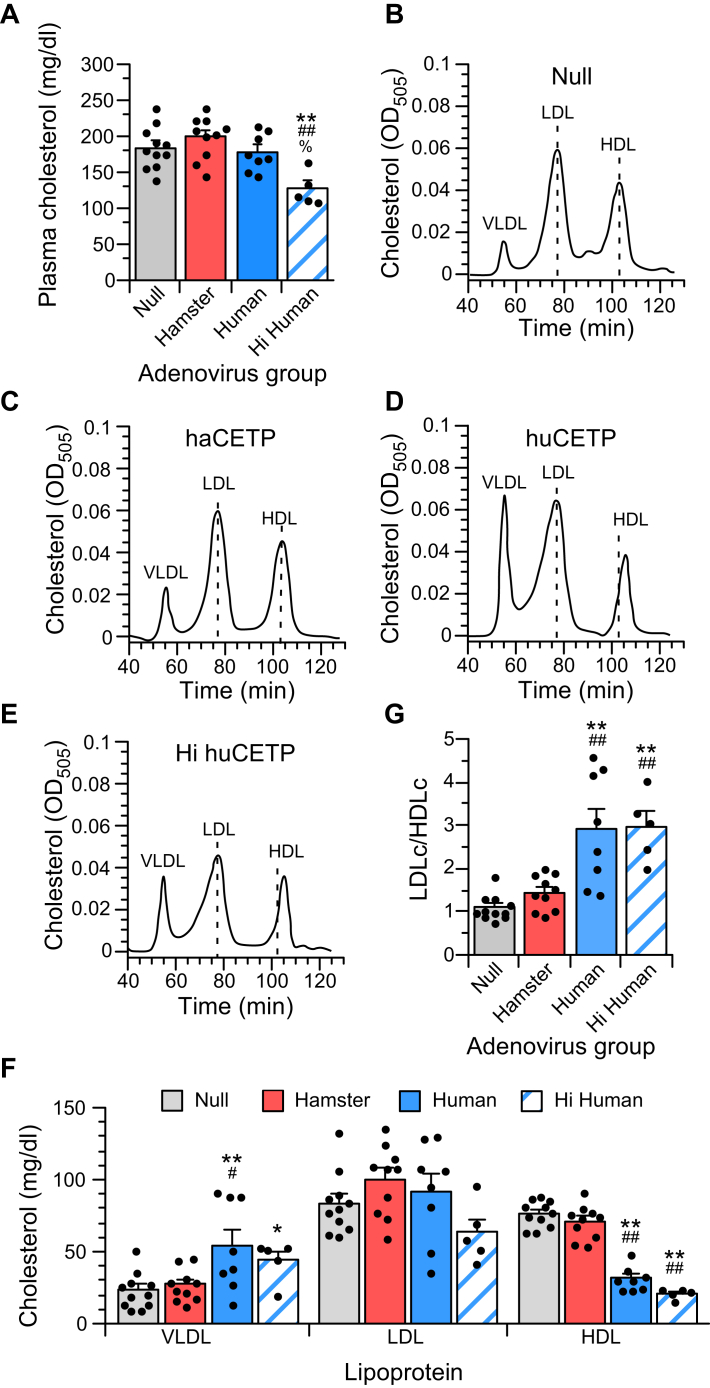
Plasma cholesterol and lipoprotein levels in fat/cholesterol-fed hamsters with altered CETP expression. A: Plasma cholesterol concentrations. B–E: Typical FPLC cholesterol profile for null (B), haCETP (C), haCETP (D), and Hi huCETP (E) animals. Dashed lines in each profile show the peak retention time for null LDL and HDL. F: Lipoprotein cholesterol concentrations in the plasma of the indicated adenovirus group. G: LDL/HDL ratios. Values are mean ± SEM of null (11), haCETP (10), huCETP (8), and Hi huCETP (5) animals. *∗P* < 0.05 versus null, *∗∗P* < 0.01 versus null, ^*#*^*P* < 0.05 versus haCETP, ^*##*^*P* < 0.01 versus haCETP, ^%^*P* < 0.05 versus huCETP, ^%%^*P* < 0.01 versus huCETP.

Compared with chow-fed animals (9, 21), the high-fat/cholesterol diet increases plasma cholesterol in Ad-null animals almost twofold (Fig. 1A), resulting in cholesterols levels similar to those in normolipidemic humans. Overexpression of hamster CETP, or the exogenous expression of human CETP at similar levels, did not alter total cholesterol levels (Fig. 1A). By contrast, in Hi huCETP animals, total plasma cholesterol levels were decreased by more than 50 mg/dl.

Hamster plasmas were fractionated by gel filtration FPCL to quantify the distribution of cholesterol among lipoproteins. Representative profiles are shown in Fig. 1B–E. The retention times for LDL and HDL in animals overexpressing hamster CETP were the same as those for Ad-null controls [Fig. 1B, C (note dashed lines)]. In animals expressing human CETP, although the peak retention time for LDL was the same as control LDL, the shape of the LDL peak was altered (Fig. 1D, E). In Ad-null and Ad-haCETP plasmas, the LDL peak was almost symmetrical, whereas with LDL from huCETP animals the LDL peak was broader and skewed to the left, indicating increased levels of larger particles. On the other hand, HDL from huCETP animals consistently eluted at a later time than in the control, indicating a smaller size.

The overall distribution of cholesterol among lipoproteins in each group is shown in Fig. 1F. In haCETP animals, the overexpression of hamster CETP had no effect on the concentrations of VLDL, LDL, or HDL or on the ratio of LDLc to HDLc (Fig. 1G). In contrast, in huCETP animals the expression of human CETP markedly decreased HDLc and increased VLDLc twofold without a change in LDL cholesterol. This caused an almost threefold rise in the LDLc/HDLc ratio. The dramatically lower total plasma cholesterol in animals expressing high levels of human CETP was primarily due to a further decrease in HDLc levels. Even though LDLc was not statistically reduced in Hi huCETP animals, the ratio of LDLc to HDLc was the same as in animals expressing lower levels of human CETP.

For both haCETP and huCETP groups, altered CETP expression had small or no effect on plasma levels of VLDL or LDL protein (Table 2). Therefore, the increased VLDL cholesterol noted above in animals expressing human CETP arises from an increase in the cholesterol content of VLDL instead of increased particle number. On the other hand, HDL protein levels were reduced by ∼50 mg/dl in huCETP animals compared with null. And in animals expressing higher levels of human CETP, HDL protein was reduced by more than 120 mg/dl.

**Table 2:** Lipoprotein protein concentrations

Ad Group	VLDL	LDL	HDL
	mg Protein/dl		
Null	25.5 ± 3.2	68.5 ± 3.7	232.8 ± 3.2
Hamster	20.8 ± 2.1	67.4 ± 6.1	209.4 ± 13.6
Human	34.6 ± 5.2^c^	90.5 ± 9.2	183.8 ± 10.1^a^
Hi Human	19.0 ± 1.0	58.4 ± 7.1^e^	109.5 ± 10.4^b^^,^^d^^,^^f^

Ad, adenovirus.

The plasma protein concentrations of lipoproteins were calculated from the plasma cholesterol concentration of each lipoprotein fraction, determined by FPLC, times the ratio of protein to cholesterol in each lipoprotein fraction determined on lipoproteins isolated from plasma by ultracentrifugation. Values are mean ± SEM of the group sizes shown in Table 1.

a *P* < 0.05 versus null.

b *P* < 0.01 versus null.

c *P* < 0.05 versus haCETP.

d *P* < 0.01 versus haCETP.

e *P* < 0.05 versus huCETP.

f *P* < 0.01 versus huCETP.

### Lipoprotein compositional changes caused by CETP expression

To further investigate the effects of CETP overexpression on lipoproteins, LDL and HDL were isolated by ultracentrifugation and chemically characterized. Although LDLc levels were not affected (Fig. 1), overexpression of hamster CETP increased the ratio of CE/TG in LDL twofold (Table 3). Compared with haCETP LDL, huCETP LDL contained less CE, more TG, and a reduced CE/TG ratio. High-level expression of human CETP further enriched LDL in TG. This also led to a decrease in the ratio of surface to core components, consistent with the increase in LDL size observed on gel filtration. Overall, hamster and human CETP have opposite effects on the CE versus TG content of LDL.

**Table 3:** Lipid composition of LDL and HDL

Lp	Ad Group	FC	CE	TG	PL	CE/TG	S/C
		μg/mg Protein					
LDL	Null	455 ± 20	1,320 ± 96	467 ± 50	1,277 ± 36	3.17 ± 0.39	1.57 ± 0.09
	haCETP	480 ± 16	1760 ± 140	319 ± 40	1,288 ± 23	6.55 ± 1.10^a^	1.37 ± 0.07
	huCETP	337 ± 28 b^,^^d^	1,188 ± 211^c^	668 ± 98^d^	1,216 ± 70	2.52 ± 0.85^d^	1.33 ± 0.12
	Hi huCETP	346 ± 19 a^,^^d^	1,251 ± 107	977 ± 55 b^,^^d^^,^^e^	1,160 ± 29	1.30 ± 0.14^d^	1.14 ± 0.06^a^
HDL	Null	48 ± 4	487 ± 26	1.6 ± 0.4	758 ± 21	411 ± 54	3.60 ± 0.18
	haCETP	47 ± 5	509 ± 55	2.0 ± 0.4	738 ± 73	336 ± 68	3.43 ± 0.37
	huCETP	25 ± 6 b^,^^c^	260 ± 33 b^,^^d^	26 ± 8^b^^,^^d^	657 ± 35	20.6 ± 8.2 b^,^^d^	6.04 ± 0.55 b^,^^d^
	Hi huCETP	25 ± 2 a^,^^c^	292 ± 20 a^,^^d^	53 ± 8 b^,^^d^^,^^f^	608 ± 18	5.9 ± 0.9 b^,^^d^	4.65 ± 0.32

Ad, adenovirus; Lp, lipoprotein.

The free cholesterol (FC), cholesteryl ester (CE), triglyceride (TG), and phospholipid (PL) contents of LDL and HDL were quantified as described in the Methods. The ratio of components residing in the LDL surface (S) and core (C) was calculated as: (Protein + PL + FC)/(CE+TG). For HDL, this calculation assumed 40% of FC resides in the core (30). Mean ± SEM of the group sizes shown in Table 1.

a *P* < 0.05 versus null.

b *P* < 0.01 versus null.

c *P* < 0.05 versus haCETP.

d *P* < 0.01 versus haCETP.

e *P* < 0.05 vs huCETP.

f *P* < 0.01 vs huCETP.

In contrast to LDL, overexpression of hamster CETP had no effect on HDL lipid composition (Table 3). However, HDL from huCETP animals had reduced CE and elevated TG content with a greatly decreased CE/TG ratio. The markedly reduced ratio of surface to core components in these HDLs also show they are much smaller. Excess human CETP expression further increased the TG content of HDL. Overall, this compositional analysis shows that hamster CETP overexpression only affects LDL, whereas expressing human CETP causes larger changes in lipoprotein composition, with HDL being the primary target.

Changes in HDL size were further assessed by native polyacrylamide gel electrophoresis. Consistent with compositional analysis, hamster CETP overexpression had no effect on the distribution of HDL among five size subfractions (Table 4). huCETP HDL particles were smaller, with pronounced decreases in HDL_2_ sized particles and increases in HDL_3_ particles (Table 4), resulting in a 3.6-fold increase in the HDL_3_/HDL_2_ ratio compared with null HDL. This pattern of change was also seen when HDL subfraction levels were quantified by their plasma protein concentration instead of their percent distribution (not shown). In Hi huCETP animals, the higher expression of human CETP had comparatively small additional effects on the size distribution of HDL. HDL_2a_ levels were decreased, and HDL_3b_ and _3c_ were increased, causing a further rise in the ratio of HDL_3_/HDL_2_. Thus, although high levels of human CETP expression dramatically reduce plasma HDL cholesterol (Fig. 1) and protein levels (Table 2), this reduction largely occurs without major changes in the size distribution of HDL particles.

**Table 4:** HDL size distribution

Ad Group	Protein Distribution (%)					
	HDL2b	HDL2a	HDL3a	HDL3b	HDL3c	HDL3/HDL2
Null	45.5 ± 1.1	28.0 ± 0.9	19.9 ± 0.9	5.2 ± 0.3	1.5 ± 0.2	0.36 ± 0.02
haCETP	40.8 ± 1.3	28.2 ± 0.6	21.8 ± 1.0	7.1 ± 0.7	2.3 ± 0.3	0.46 ± 0.04
huCETP	20.8 ± 2.1^a^^,^^c^	24.4 ± 2.3	30.4 ± 1.3^a^^,^^c^	17.2 ± 2.1^a^^,^^c^	7.3 ± 1.0^a^^,^^c^	1.30 ± 0.16^a^^,^^c^
Hi huCETP	17.6 ± 0.5^a^^,^^c^	17.6 ± 0.6^a^^,^^c^^,^^d^	26.6 ± 1.2^a^^,^^b^	24.2 ± 1.3^a^^,^^c^^,^^e^	14.1 ± 0.8^a^^,^^c^^,^^e^	1.85 ± 0.08^a^^,^^c^^,^^e^

Ad, adenovirus.

HDL was isolated from hamsters fed a high-fat/cholesterol diet. HDL was fractionated by nondenaturing gradient gel electrophoresis. Shown are the percentages of total HDL protein in each subfraction. Values are mean ± SEM of the group sizes shown in Table 1

a *P* < 0.01 versus null.

b *P* < 0.05 versus haCETP.

c *P* < 0.01 versus haCETP.

d *P* < 0.05 versus huCETP.

e *P* < 0.01 versus huCETP.

### Effect of modified composition on LDL function

Expression of hamster and human CETP altered the lipid composition of LDL, and human CETP expression increased the abundance of larger LDL particles. To assess whether these changes alter interaction with the hepatic LDL receptor, LDLs were labeled with the residualizing fluorophore, DiI. Studies of the dose-dependent uptake of DiI-LDL in HepG2/C3A cells were performed to determine *Km* and *V*_*max*_ values for each LDL. *Km* values for LDL isolated from null (22.6 ± 3.3 μg LDL protein/ml), haCETP (21.2 ± 1.2), huCETP (24.7 ± 3.7), and Hi huCETP (22.5 ± 2.0) animals were not different. *V*_*max*_ values for LDL isolated from each of the experimental groups were greater than or equal to that of null LDL [2.96 ± 0.17 (null), 3.51 ± 0.07 (haCETP), 3.58 ± 0.22 (huCETP), and 2.90 ± 0.15 (Hi huCETP)].

### Consequences of CETP-driven modification of HDL

HDLs have multiple functions in cholesterol homeostasis. The large compositional and size changes induced by human CETP expression suggest some of these basic HDL functions may be altered in these animals.

**Fig. 2:**
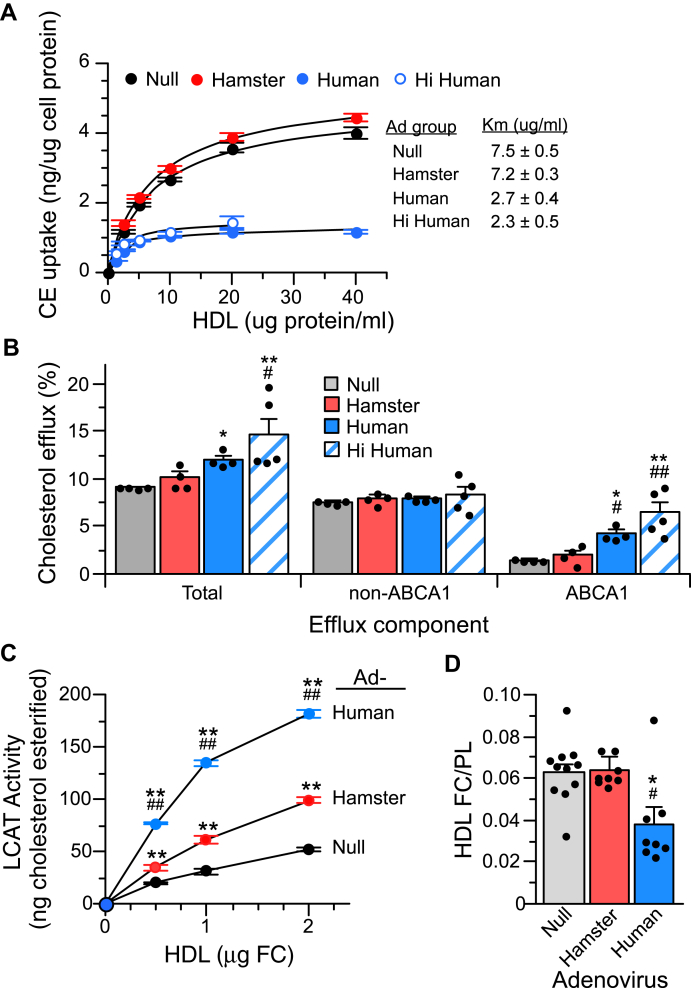
Functional properties of HDL. A: SRBI-mediated uptake of HDL CE. HDLs from four to six animals were pooled for each adenovirus group and labeled with ^3^H-CEth. Shown is the dose-dependent uptake of ^3^H-CEth by SRBI-expressing CHO cells (mean ± SEM, n = 6 for each concentration except n = 3 for Hi huCETP HDL). All huCETP and Hi huCETP data points are *P* < 0.01 versus null and haCETP. B: HDL-mediated free cholesterol (FC) efflux from macrophages. RAW 267.4 macrophages prelabeled with ^3^H-FC were incubated with HDL (50 μg protein). ABCA1-dependent and -independent pathways were determined from cells incubated with ± cAMP as described in Methods. Values are mean ± SEM of separate measurements made on HDL from four animals in each group, each assayed in triplicate. C: Capacity of HDL to support LCAT activity. For each adenovirus group, HDLs isolated from three animals were pooled, labeled with ^3^H-FC, and assayed for their ability to support LCAT activity from an exogenous source. FC esterification due to LCAT coisolated with the HDL has been subtracted. Values are mean ± SD of triplicate determinations on each pool. D: HDL-free cholesterol content. Values are mean ± SEM. See Fig. 1 legend for D group sizes. See Fig. 1 legend for *P* value symbol definitions.

HDL delivers CE to cells by a selective uptake mechanism involving the SRBI receptor (31, 32). To measure CE uptake, HDLs were labeled with ^3^H-CEth, a nondegradable CE analogue. Dose-dependent CE uptake studies were performed in LDL receptor-deficient, CHO cells expressing the mouse SRBI receptor. Overexpression of hamster CETP did not alter the ability of HDL from these animals to deliver CE to cells (Fig. 2A). In contrast, the apparent *Km* for HDL isolated from animals expressing human CETP was much lower, indicating higher-affinity interactions. However, these HDLs were ineffective in delivering ^3^H-CEth to cells. Since only half of the reduced CE uptake from huCETP and Hi huCETP HDL can be explained by the lower CE content of these particles (Table 3), it appears that the small HDL particles present in animals expressing human CETP are inefficient donors of CE to cells during the selective uptake process.

The cholesterol efflux potential of HDL was determined from the extent of ^3^H-FC efflux from labeled RAW macrophages. Cholesterol efflux mediated by ApoB-depleted serum from haCETP and huCETP animals was not different from that of null animals (supplemental Table S2). Cholesterol efflux by ApoB-depleted serum from Hi huCETP animals was increased 35%. Since huCETP and Hi huCETP serum contain significantly less HDL (Fig. 1), these findings suggest that the HDL in these sera more effectively promotes cholesterol efflux. Indeed, when compared on an equal protein basis, huCETP HDL facilitated greater cholesterol efflux (Fig. 2B). This enhanced capacity was due to a threefold increase in ABCA1-dependent efflux compared with Ad-null HDL. HDL from Hi huCETP animals supported even greater total cholesterol and ABCA1-dependent cholesterol efflux. Overexpression of hamster CETP did not alter the cholesterol efflux potential of HDL.

To assess the capacity of HDL to support LCAT activity, HDLs were labeled with ^3^H-FC and then the extent to which this cholesterol was esterified by a constant level of exogenous LCAT was measured. haCETP HDL supported twofold greater LCAT activity than Ad-null HDL, even though its general physicochemical properties were only modestly altered by hamster CETP overexpression (Fig. 2C). Cholesterol esterification supported by huCETP HDL was up to 4.3-fold higher than null HDL. The increased capacity of HDL from human CETP animals to support the conversion of FC to CE in vitro is consistent with the reduced FC content of HDL (Fig. 2D) and LDL [FC/PL = 0.357 ± 0.014 (null), 0.373 ± 0.013 (haCETP), and 0.276 ± 0.012 (huCETP, *P*< 0.01 vs null and haCETP)] from these animals.

### Gene expression in response to altered lipoprotein functional properties

Hamsters consuming a high-fat/cholesterol diet have marked changes in the expression of multiple hepatic genes involved in lipid metabolism (10). The altered cholesterol content and modified functional properties of lipoproteins in animals expressing human CETP may further impact the expression of these genes. To evaluate this, the expression of genes involved in lipid metabolism that are either suppressed (*LDLR*, *HMGCR*, *SCARB1*) (33, 34) or stimulated (*CYP7A1*, *ABCA1*, *ABCG1*) (34, 35) by cholesterol, or unresponsive (*MTTP*, *SREBF2*) (36, 37, 38) to cholesterol, was measured. Overexpression of hamster CETP did not significantly alter the expression of any of these genes (Table 5). Although hepatic expression of several genes was modified in huCETP or Hi huCETP animals, there was no consistent relationship between the genes affected and the expected response of those genes to cholesterol. These data show that, in general, the aberrant lipoproteins in animals expressing human CETP do not modify the expression of cholesterol-regulated genes beyond the set-point determined by the cholesterol-enriched diet.

**Table 5:** Expression of lipid metabolic genes in liver

Ad Group	Relative mRNA levels							
	*LDLR*	*HMGCR*	*SCARB1*	*CYP7A1*	*ABCA1*	*ABCG1*	*MTTP*	*SREBF2*
Null (8)	1.00 ± 0.07	1.00 ± 0.04	1.00 ± 0.06	1.00 ± 0.21	1.00 ± 0.15	1.00 ± 0.09	1.00 ± 0.08	1.00 ± 0.18
haCETP (9)	1.39 ± 0.20	0.94 ± 0.06	1.34 ± 0.12	0.53 ± 0.09	1.57 ± 0.36	1.19 ± 0.16	1.15 ± 0.20	1.47 ± 0.25
huCETP (8)	0.94 ± 0.12	0.74 ± 0.19	0.58 ± 0.11^a^^,^^d^	0.30 ± 0.06^b^	0.77 ± 0.10	0.90 ± 0.07	0.98 ± 0.17	0.70 ± 0.11^c^
Hi huCETP (5)	0.94 ± 0.19	1.03 ± 0.13	0.89 ± 0.20	0.44 ± 0.16	1.39 ± 0.32	1.05 ± 0.15	0.54 ± 0.09^a^	0.62 ± 0.05^c^

Ad, adenovirus.

Relative mRNA levels were determined by qPCR as described in Methods. Genes are grouped based on whether their expression is known to be decreased (LDLR, HMGCR, SCARBI), increased (CYP7A1, ABCA1, ABCG1), or unaffected (*MTTP*, *SREBF2*) by elevated cholesterol. Values are mean ± SEM of the indicated group sizes.

^e^*P* < 0.05 versus huCETP.

^f^*P* < 0.01 versus huCETP.

a *P* < 0.05 versus null.

b *P* < 0.01 versus null.

c *P* < 0.05 versus haCETP.

d *P* < 0.01 versus haCETP.

As suggested by their mRNA values, hepatic LDL receptor protein levels were not different between null, haCETP, and huCETP groups [1.01 ± 0.09 (n = 8), 0.99 ± 0.17 (n = 6) and 1.11 ± 0.16 (n = 7), respectively]. Although *SCARB1* mRNA was decreased in huCETP livers (Table 5), liver levels of its gene product, SRBI, were not altered among these groups [1.00 ± 0.09 (null), 1.14 ± 0.20 (haCETP) and 0.83 ± 0.18 (huCETP)]. These data show that neither haCETP nor huCETP expression alters hepatic levels of these principal lipoprotein receptors.

### Reverse cholesterol transport

**Fig. 3:**
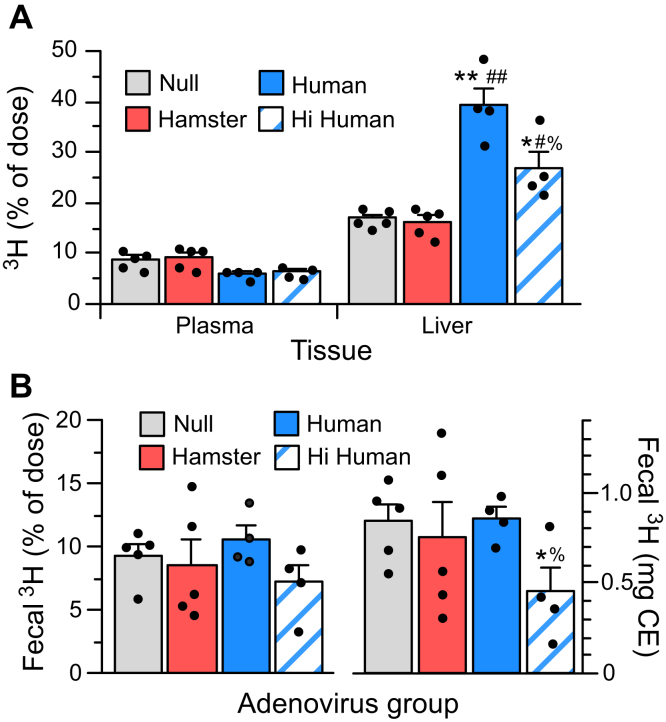
Reverse cholesterol transport (RCT). Animals received ^3^H-CE labeled HDL intravenously and were euthanized after 48 h. A: The percentage of injected ^3^H recovered in plasma and liver. B: The percent of injected ^3^H recovered in feces, and this fecal ^3^H expressed as milligram plasma CE. This fecal CE mass value was calculated as the fraction of injected ^3^H recovered in feces times the mass of CE in plasma. Note the difference in y-axis scale between A and B. Values are mean ± SEM of Ad-null (5), Ad-haCETP (5), Ad-huCETP (4), and high expressing Ad-huCETP (4) animals. See Fig. 1 legend for *P* value symbol definitions.

Reduced HDL levels and altered functional properties of HDL in huCETP and Hi huCETP animals suggest that cholesterol RCT may be altered. The RCT of HDL-associated CE into feces was measured in hamsters 48 h after the injection of ^3^H-CE-labeled HDL. In animals expressing human CETP, there was a modest reduction in the percentage of ^3^H remaining in plasma at the end of the RCT assay, although this did not reach statistical significance (*P* = 0.053) (Fig. 3A). Most notably, the percentage of injected ^3^H recovered in the liver of animals expressing human CETP was increased up to 2.3-fold. The percentage of ^3^H present in the feces was unchanged (Fig. 3B). Since plasma CETP equilibrates the injected ^3^H-CE originally in HDL among all lipoproteins during the RCT experiment, a more appropriate calculation of CE delivery to feces takes into account the total plasma CE pool size. Calculated this way, very high levels of human CETP expression reduced fecal cholesterol excretion by 40%, but huCETP animals with CETP levels similar to those of haCETP animals did not have altered fecal excretion (Fig. 3B).

### Liver cholesterol and bile acid content

**Fig. 4:**
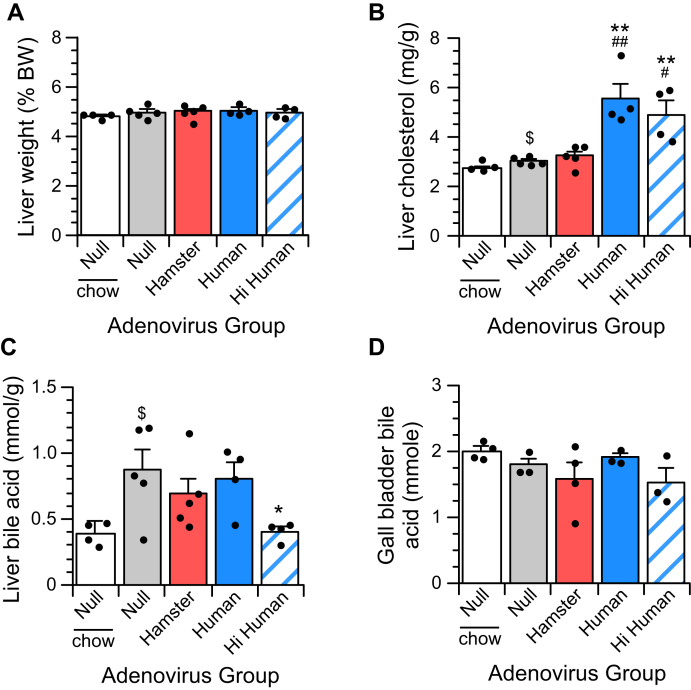
Liver sterol content. Animals consumed a high-fat/cholesterol diet for 6 days prior to analysis, unless indicated otherwise. Animals on this high-fat diet weighed 116.8 ± 1.5 g (null), 117.9 ± 2.2 g (haCETP), 117.7 ± 2.1 g (huCETP), and 120.1 ± 5.1 g (Hi huCETP) at the end of the study. Liver weights are based on tissue wet weight. Livers were assayed for cholesterol and bile acid as described in Methods. Data for null animals consuming a chow diet have been, in part, previously published (9). Values are mean ± SEM (n = 4–5 animals per group). *∗P* < 0.05 versus null, *∗∗P* < 0.01 versus null, ^*#*^*P* < 0.05 versus haCETP, ^*##*^*P* < 0.01 versus haCETP, ^%^*P* < 0.05 versus huCETP, ^%%^*P* < 0.01 versus huCETP. Null animals consuming chow or high-fat/cholesterol diets were compared separately by *t*-test - ^$^*P* < 0.05 versus chow null. BW, body weight.

RCT data showing an increased accumulation of HDL-derived ^3^H-CE in the livers of animals expressing human CETP suggest that hepatic cholesterol may be increased in these animals. Compared with chow-fed animals, the liver size was not affected by the high-fat/cholesterol diet (Fig. 4A). Among animals consuming the fat-enriched diet, liver size was unchanged by altered CETP expression. It is surprising that, among control animals consuming chow or high-fat/cholesterol diet, the cholesterol-enriched diet only increased liver cholesterol by 10% (Fig. 4B). This elevated cholesterol content was unaffected by overexpression of hamster CETP. However, the cholesterol content of livers from animals expressing human CETP was increased up to 1.8-fold regardless of the extent to which human CETP was expressed.

Peripheral cholesterol is delivered to the liver for excretion in bile. Levels of bile acids in the liver are tightly regulated to prevent toxicity and to maintain a robust mechanism for cholesterol excretion. We measured the bile acid content of liver and isolated gall bladder to assess the function of this cholesterol excretion pathway. The bile acid content of livers from null, haCETP, and huCETP animals consuming the fat/cholesterol diet were not different, but it was decreased by 50% in animals expressing high levels of human CETP (Fig. 4C). This decrease in liver bile acids mirrors the reduced plasma cholesterol levels in these animals. Gall bladder bile levels were not changed by diet or altered CETP expression (Fig. 4D). The absence of apparent cholestasis in animals expressing human CETP suggests the cycling of bile acids through the enterohepatic system is not impaired (39) and is not likely the cause of the elevated cholesterol levels in the livers of these animals.

## Discussion

Our previous studies in chow-fed hamsters demonstrated that the physical properties of HDL could be modified by increasing the preference of plasma CETP for CE rather than TG as a substrate (9). Compared with humans, chow-fed hamsters have low LDL and low total plasma cholesterol and HDL is the major cholesterol-carrying lipoprotein. Also, VLDL levels are very low. Low VLDL constrains plasma CETP activity in vivo by limiting the availability of TG for the CETP-mediated heteroexchange of CE and TG (7, 8). To better understand the potential impact of modifying CETP's substrate preference on lipoprotein metabolism under conditions more similar to those of humans, hamsters were fed a diet containing 0.12% cholesterol and 20% hydrogenated coconut oil. In these animals, plasma VLDL is increased four- to fivefold (10), LDL is the predominant cholesterol-carrying plasma lipoprotein, and total plasma cholesterol levels are similar to those in normolipidemic humans.

Overexpression of hamster CETP in fat-fed animals had no effect on the plasma levels of individual lipoproteins. Furthermore, although LDLs were enriched in CE relative to TG, there was no change in HDL lipid composition, LDL and HDL size, or HDL function. This is similar to that seen in chow-fed animals overexpressing hamster CETP (9), Thus, even with the increased levels of VLDL in fat-fed animals to promote CETP activity in vivo, overexpression of CETP has little consequence.

In fat-fed animals, the effect of human CETP expression fell into two categories. In the first, essentially all of the modifications in LDL and HDL lipid composition, reduced HDL levels and particle size, and increased VLDL cholesterol previously observed in chow-fed, human CETP animals were magnified by the high-fat diet. The low HDL levels in these animals may be secondary to the large reduction in HDL particle size, which increases the turnover of HDL components in the kidney (40, 41). Second, human CETP expression in fat-fed animals elicited novel phenotypes. HDLs from animals expressing human CETP were much better substrates for LCAT and promoted greater FC efflux from cells via an ABCAI-dependent mechanism. However, these HDLs were also ineffective in delivering CE to cells via SRBI. Expression of human CETP in fat-fed animals also caused hepatic cholesterol levels to increase almost twofold. This cholesterol enrichment was the same regardless of the extent to which human CETP was expressed. It is notable that hepatic cholesterol levels in Ad-null animals were minimally increased by the fat/cholesterol-enriched diet itself, showing a robust capacity to maintain cholesterol homeostasis during short-term cholesterol feeding. It appears that human CETP expression in animals consuming a cholesterol-enriched diet disrupts mechanisms that normally control hepatic cholesterol levels.

The mechanistic basis for the enhanced hepatic cholesterol content of animals expressing human CETP is unclear. Gene expression analysis did not identify changes in mRNA levels of regulatory genes in the cholesterol or bile acid synthetic pathways or in receptors involved in LDL or HDL metabolism that might contribute to increased hepatic cholesterol. And, LDL or HDL from huCETP animals did not promote increased cholesterol delivery to cells. However, human CETP expression does stimulate the transfer of CE into VLDL. Hepatic uptake of these cholesterol-enriched VLDLs, or their remnants, may drive hepatic cholesterol accumulation. This remains to be tested experimentally. Alternatively, several studies have shown that human CETP also functions inside cells to control the accumulation of CE and TG in lipid droplets (11, 42, 43). The expression of human CETP may modify this process in the livers of fat-fed hamsters causing cholesterol to accumulate. Owing to their different preferences for CE versus TG as substrate, human and hamster CETP may uniquely impact cellular lipid storage.

In chow-fed animals, overexpressing hamster CETP or expressing human CETP did not modify the extent to which HDL-derived CE was excreted into feces during the RCT assay (9). Consumption of a fat-enriched diet did not change this, even though HDLs in huCETP animals are less abundant, more modified, and functionally altered. In fat-fed hamsters expressing very high human CETP levels, excretion of cholesterol derived from HDL was reduced, perhaps due to a marked reduction in both LDL and HDL levels. The maintenance of effective cholesterol excretion even when HDL levels are reduced is consistent with kinetic studies in other CETP-expressing species showing that the vast majority of plasma CE recovered in bile is derived from VLDL and LDL, not HDL (44). It is important to note that the expression of human CETP in fat-fed animals caused a large portion of HDL-derived CE to remain in the liver during the RCT assay. It is notable that the extent of this accumulation is similar to the increase in cholesterol mass in the livers of these animals. If the ^3^H-cholesterol delivered to the liver during the RCT assay is diluted by this larger hepatic cholesterol pool prior to excretion, then estimates of cholesterol excretion based on ^3^H will be underestimated. For this reason, the actual reverse transport of HDL-derived CE into the feces of huCETP animals may actually exceed that of control animals. Studies using methods that can distinguish the contributions of dietary versus biliary and nonbiliary excretion pathways to the fecal sterol pool are needed to resolve this issue.

A striking feature of human CETP expression is its almost exclusive impact on HDL regardless of animal diet. Human and hamster lipoproteins are equivalent substrates for human CETP in vitro (9), so it is unlikely that there is a unique interaction between human CETP and hamster HDL. There are at least two other factors that may contribute to the preferential impact on HDL. First, HDL is more metabolically active than LDL. HDL is the point of entry for peripheral cholesterol and its conversion to CE. Thus, the relatively short time course of this study may bias observations toward more metabolically active lipoproteins. Second, the HDL-centric effects of human CETP expression may arise because HDL is a major site of CETP activity in plasma. This is not due to a preferred interaction between CETP and HDL per se, but rather due to the effects of apolipoprotein F (ApoF). ApoF blocks CETP activity on LDL and enhances lipid transfers involving HDL (10, 45). We have observed that plasma ApoF levels are not changed by human CETP expression (unpublished), showing that the preferential transfer of lipids in HDL likely persists in these animals. Separately, or in combination, these factors may favor the remodeling of HDL in animals where the substrate preference of CETP is altered.

In summary, expressing human CETP in fat-fed hamsters, which have a more human-like lipoprotein profile, results in larger changes and a more diverse lipid phenotype than occurs in chow-fed animals expressing this protein. Regardless of diet, HDL is the primary site for modifications caused by human CETP, which results in decreased plasma HDL levels. Uniquely, human CETP expression in fat-fed animals causes cholesterol accumulation in the liver, suggesting a major change in cholesterol processing in these animals. These studies further demonstrate the capacity of CETP to control lipoprotein and lipid metabolism. Altering the substrate specificity of CETP for CE versus TG provides a powerful tool for modulating HDL metabolism and sterol balance in vivo.

### Data availability

All data supporting the findings of this study are contained in the article or the supplemental data.

## Conflict of interest

The authors declare that they have no conflicts of interest with the contents of this article.
