# Supplementary material for: The lipid substrate preference of CETP controls the biochemical properties of HDL in fat/cholesterol-fed hamsters
Source: J Lipid Res. 2021 Jan 27;62:100027. doi: 10.1016/j.jlr.2021.100027 (PMC7933494; doi:10.1016/j.jlr.2021.100027)
Supplement: Supplemental Tables S1 and S2 [file mmc1.docx]

**Supplemental Material**

The lipid substrate preference of CETP controls the biochemical properties of HDL in fat/cholesterol-fed hamsters

Richard E. Morton, Daniel Mihna, and Yan Liu

**Supplemental Table S1**

qPCR primers for the indicated golden Syrian hamster (*Mesocricetus auratus*) gene. The *SREBF2* primers were reported by Dong, B. et al. (J. Biol. Chem. 290: 4047-4058, 2015).

| Gene | Forward primer 5’ to 3’ | Reverse primer 5’ to 3’ |
| --- | --- | --- |
| *ABCA1* | GTGCTTGTGACCCTTGGAAC | TGGGATTGTCAGAGGTTTGGG |
| *ABCG1* | TGACACAATCCCAGCCTACC | GATGACTCCCTCAAAGCCGT |
| *CYP7A1* | TACTAGATAGCATCATCAAGGAGGCTC | CCATCCTCAAGGTGCAGAGTG |
| *HMGCR* | GCTAGGTGTTCAAGGAGCGT | CCACACACAATTCGGGCAAG |
| *LDLR* | AAGGCAGCTACAAGTGCGAG | TTCCTTACCTCGTGGCGATT |
| *MTTP* | AGAGGAAAACCTGGACTCCTATG | AGCATTTTGGACATCAGATCACT |
| *SCARB1* | ATGCCCTCGCTCATCAAACA | CCCGCACTATTGGCTTCTCA |
| *SREBF2* | GAGAGCTGTGAATTTTCCAGTG | CTACAGATGATATCCGGACCAA |
| *ACTB* | GTGGATCAGCAAGCAGGAGT | CTCAGTAACAGTCCGCCTAGAA |

**Supplemental Table S2**

Free cholesterol (FC) efflux mediated by apolipoprotein B-depleted serum. RAW 267.4 macrophages prelabeled with ^3^H-FC were incubated with media containing 2.8% apolipoprotein B-depleted serum (= 2% of original serum) as described in the Methods. ABCA1-dependent and -independent pathways were determined from cell incubated with ± 8-bromo cAMP. Values are the mean ± SEM of values from the indicated number of samples. *^a^* – p< 0.05 vs null, *^b^* – p< 0.01 vs null, *^c^* – p< 0.05 vs hamster. Ad, Adenovirus.

| Ad group | Total efflux | ABCA1 independent | ABCA1 dependent |
| --- | --- | --- | --- |
| Null (4) | 11.9 ± 0.8 | 6.9 ± 0.7 | 5.0 ± 0.7 |
| haCETP (4) | 12.4 ± 1.0 | 6.6 ± 0.5 | 5.6 ± 0.7 |
| huCETP (4) | 12.2 ± 0.8 | 5.4 ± 0.4 | 6.9 ± 0.7 |
| Hi huCETP (5) | 16.3 ± 1.3*^a^* | 6.5 ± 0.4 | 9.8 ± 1.1*^b,c^* |
